# m6A Regulator-Associated Modification Patterns and Immune Infiltration of the Tumor Microenvironment in Hepatocarcinoma

**DOI:** 10.3389/fcell.2021.687756

**Published:** 2021-07-02

**Authors:** Jianhao Li, Weiwei Wang, Yubing Zhou, Liwen Liu, Guizhen Zhang, Kelei Guan, Xichun Cui, Xin Liu, Maoxin Huang, Guangying Cui, Ranran Sun

**Affiliations:** ^1^Precision Medicine Center, The First Affiliated Hospital of Zhengzhou University, Zhengzhou, China; ^2^Key Laboratory of Clinical Medicine, The First Affiliated Hospital of Zhengzhou University, Zhengzhou, China; ^3^Department of Pathology, The First Affiliated Hospital of Zhengzhou University, Zhengzhou, China; ^4^Department of Pharmacy, The First Affiliated Hospital of Zhengzhou University, Zhengzhou, China; ^5^Department of Dermatology, The First Affiliated Hospital of Zhengzhou University, Zhengzhou, China

**Keywords:** N6-methyladenosine, hepatocellular carcinoma, tumor microenvironment, prognosis, immune infiltration

## Abstract

**Background:** Immunotherapy elicits durable responses in many tumors. Nevertheless, the positive response to immunotherapy always depends on the dynamic interactions between the tumor cells and infiltrating lymphocytes in the tumor microenvironment (TME). Currently, the application of immunotherapy in hepatocellular carcinoma (HCC) has achieved limited success. The ectopic modification of N6-methyladenosine (m6A) is a common feature in multiple tumors. However, the relationship between m6A modification with HCC clinical features, prognosis, immune cell infiltration, and immunotherapy efficacy remains unclear.

**Materials and Methods:** Here, we comprehensively evaluated m6A modification clusters based on 22 m6A regulators and systematically explored the relationship between m6A modification with tumor progression, prognosis, and immune cell infiltration characteristics. The m6Ascore was calculated by principal component analysis to quantify the m6A modifications of individual patients. Key regulators involved in immunoregulation in HCC were identified using immunohistochemistry and immunofluorescence.

**Results:** Three distinct m6A modification clusters were identified. The m6A clusters were significantly associated with clinical features, prognosis, and immune cell infiltration. The three clusters were highly consistent with the three tumor immune phenotypes, i.e., immune-excluded, immune-inflamed, and immune-desert. Comprehensive bioinformatics analysis revealed that high m6Ascore was closely associated with tumor progression, poor prognosis, and immunotherapy non-response. m6A regulators were dysregulated in HCC tissues. Hence, they play a role as predictors of poor prognosis. Tissue microarray demonstrated that overexpressed YTHDF1 was associated with low CD3^+^ and CD8^+^ T cell infiltration in HCC.

**Conclusion:** Our findings demonstrate that m6A modification patterns play a crucial role in the tumor immune microenvironment and the prognosis of HCC. High YTHDF1 expression is closely associated with low CD3^+^ and CD8^+^ T cell infiltration in HCC.

## Introduction

Hepatocellular carcinoma (HCC) is one of the major causes of cancer-related mortality worldwide and accounts for 80% of primary liver cancer (Lin et al., 2013; Siegel et al., 2018). Currently, surgical resection and percutaneous ethanol injection are the main treatment modalities for HCC (Dutta and Mahato, 2017). However, even though significant efforts have been made in HCC treatment and management, the 5-year overall survival (OS) remains poor, and has been attributed to late diagnosis, tumor recurrence, and unsatisfactory treatment (Forner et al., 2018; Yang and Heimbach, 2020). Therefore, it is imperative to develop powerful diagnostic and novel therapeutic strategies to improve the outcome of HCC.

N6-methyladenosine (m6A) is an RNA post-transcriptional modification that is most abundant in mammalian mRNA (Zaccara et al., 2019). m6A methylation is mediated by several proteins, which are categorized into three types: writers are methyltransferases, including WTAP, KIAA1429, RBM15, RBM15B, and METTL3/14/16; erasers such as FTO and ALKBH5, which are the only two identified m6A demethylases; and final function executions (readers) that include HNRNPs, YTHDF1/2/3, YTHDC1/2, IGF2BP1/2/3, and EIF3A (Chen et al., 2019; Zhen et al., 2020). Increasing evidence has identified the important roles m6A modifications play in various cellular processes and in cancer progression through regulating RNA stability, mRNA splicing and translation, and microRNA processing (Gilbert et al., 2016; Zhao et al., 2017). Meanwhile, m6A modification dysregulation has been correlated with tumor malignant progression and immunomodulatory abnormality (Shulman and Stern-Ginossar, 2020). Wang et al. (2019) revealed that upregulated METTL3 promoted dendritic cell (DC) activation and maturation, and that METTL3 downregulation inhibited T cell activation and aggregation. Han et al. (2019) demonstrated that inhibiting YTHDF1 enhanced CD8^+^ T cell tumor infiltration and immunotherapy efficacy in a murine tumor model. However, the specific mechanism of m6A involvement in the malignant progression and immune response of HCC remains unclear.

In the present study, we integrated the information on mRNA and protein levels of m6A regulators to comprehensively evaluate the effect of m6A modification variation on HCC malignant progression, prognosis, and immune response. Supplementary Figure 1 shows the overall study design. First, the mRNA and protein expression levels of m6A regulators in HCC were systematically explored via The Cancer Genome Atlas (TCGA) database, Gene Expression Omnibus (GEO) and a tissue microarray analysis (TMA) cohort. Then, we identified three distinct m6A modification patterns of HCC and evaluated the clinical features, prognosis value, potential mechanism, and immune infiltration of the resultant m6A clusters. Further, we explored the correlation among the YTHDF1 level, activated tumor-infiltrating lymphocytes, and related biological processes in HCC using immunohistochemistry (IHC), immunofluorescence, and comprehensive bioinformatics analysis. We reveal that m6A modification patterns play a critical role in the malignant progression and efficacy of immunotherapy in HCC.

## Materials and Methods

### Data Source

The RNA-seq transcriptome data and corresponding clinicopathological information of 370 HCC and 50 normal tissues were obtained from TCGA liver hepatocellular carcinoma cohort (TCGA-LIHC)^1^, 203 HCC and 175 normal tissue samples from the International Cancer Genome Consortium Liver Cancer-RIKEN-JP cohort (ICGC-LIRI-JP)^2^ were downloaded. GSE36376 (non-tumor = 193, tumor = 240) and GSE76297 (non-tumor = 52, tumor = 153) were gathered through the GEO database^3^, GEO) (Chaisaingmongkol et al., 2017; Cho et al., 2020).

### Unsupervised Clustering for Twenty-Two m6A Regulators

A total of 22 m6A regulator genes were curated and analyzed to identify different m6A modification patterns based on previous literature. The 22 m6A regulators genes included seven writers (WTAP, KIAA1429, RBM15, RBM15B, and METTL3/14/16), 12 readers (HNRNPs, YTHDF1/2/3, YTHDC1/2, IGF2BP1/2/3, and EIF3A), and two erasers (ALKBH5 and FTO). To ensure clustering reproducibility of our approach, we selected TCGA-LIHC (training set) and ICGC-LIRI-JP (validation set) with high heterogeneity for further analysis. The HCC Patients without follow-up information were deleted. Eventually, 367 patients from TCGA-LIHC dataset and 203 patients from ICGC-LIRI-JP database were enrolled for subsequent analysis. Supplementary Table 1 presents the detailed clinical-pathological information of TCGA and ICGC cases selected for testing database and validation set. Then, we performed unsupervised clustering to identify distinct m6A modification patterns based on the expression of 22 m6A regulators. The R package “ConsensusClusterPlus” were used to conduct the above steps and 1000 times repetitions for guaranteeing the stability of clustering. The optimal number of clusters was determined according to the consensus clustering algorithm.

### Tissue Samples

A microarray of 100 HCC tumors and adjacent normal tissue samples was constructed using a core diameter of 1.5 mm. All experiments received approvals from the Ethics Committee of the First Affiliated Hospital of Zhengzhou University.

### Immunohistochemistry and Immunofluorescent

Immunohistochemistry and immunofluorescent were performed as previously reported. Briefly (Li et al., 2019, 2020b), 5 μm thick TMA sections were deparaffinized and treated with hydrogen peroxide to quench endogenous peroxidase activity. Subsequently, the sections were incubated with related proteins antibodies at 4°C overnight. The immunoreactive cells were detected by Signal Stain^®^ DAB (CST, United States) and counterstained with Hematoxylin QS (Vector Laboratories). Two experienced pathologists who were blinded to evaluate the clinicopathological data the immunostaining samples separately and they scored the samples according to the proportion of positive cells as follows: no staining, 1+; weak staining, 2+; moderate staining, 3+; strong staining, 4+; and intense staining, 5+; The score of 1+ and 2+ was defined as low expression while the other scores were defined as high expression for statistical analysis. The CD3^+^ and CD8^+^ T cells count were performed using ImageScope (Aperio Technologies). CD3^+^ and CD8^+^ T cell density were counted as cells/mm^2^ and categorized into high and low groups. For Immunofluorescence, slides were incubated with HRP labeled second antibody. The slides were visualized with scanning laser confocal microscope and evaluated by Image-Pro Plus software. Detailed information of antibodies used in this study was showed in Supplementary Table 2.

### Single-Sample Gene Set Enrichment Analysis (ssGSEA)

Single-sample gene set enrichment analysis (ssGSEA) in R package GSVA was used to quantify the infiltration levels of the immune cell types in tumor microenvironment (TME). ssGSEA applies gene signatures expressed by immune cell populations to individual cancer samples. Supplementary Table 3 shows the detailed information of gene signatures used in this study. The deconvolution approach was used to evaluate total 24 immune cells involved in innate immunity [natural killer (NK) cells, CD56dim NK cells, CD56bright NK cells, plasmacytoid DCs, immature DCs, activated DCs, DC, neutrophils, mast cells, eosinophils, and macrophages] and adaptive immunity including B cells, CD8^+^ T cells, Cytotoxic cells, T cells, T helper cells, Tcm (central memory T cell), Tem (effector memory T cell), TFH (Follicular helper T cell), Tgd, Th1 cells, Th17 cells, Th2 cells, and Treg (Regulatory T cell).

### Gene Set Variation Analysis (GSVA) and Other Biological Pathways Analysis

The gene set variation analysis (GSVA) package was used to export the Kyoto Encyclopedia of Genes and Genomes (KEGG) pathways described in the molecular signature database and used to perform the pathway analyses of the potential mechanism of m6A clusters. Mariathasan et al. (2018) constructed a set of gene sets that stored genes associated with some biological processes, including Antigen processing and presentation (APAP), CD8 T-effector signature, Epithelial–mesenchymal transition 1 (EMT1), EMT2, EMT3, Angiogenesis, TGF-β pathway, Wnt pathway, DNA damage repair (DDR), Nucleotide excision repair (NER), DNA replication and Cell cycle (Rosenberg et al., 2016; Şenbabaoǧlu et al., 2016; Mariathasan et al., 2018). The correlation between m6A modification and other biological pathways were further explored. Supplementary Table 4 presents the detailed information of biological pathways used in this study.

### Construction of m6Ascores

To quantitatively evaluate of m6A modification patterns for individual HCC patients, we established a set of scoring system. The establishment procedures of m6A scoring system were as follows: Differential analysis and Venn diagram showed that there are 236 common differential genes among three m6A clusters. Then, we conducted the univariate Cox regression analysis for each gene. These genes with the significant prognosis were extracted for next analysis. We then performed principal component analysis (PCA) to calculate m6A score using the formula:

$$\begin{array}{c} \mathrm{m6A}\mathrm{score}=\sum \;\left({\mathrm{PC1}}_{\text{i}}+{\mathrm{PC2}}_{\text{i}}\right) \end{array}$$

where i is the expression value of each selected genes. This formula was used to calculate the m6A score for HCC patients in both the training (TCGA) and validation (ICGC) datasets.

### Statistical Analysis

All statistical analyses were conducted in R (3.5.3) statistical package unless otherwise stated. Student’s *t*-test (unpaired, two-tailed) was used to evaluate the differences between the two independent groups. One-way ANOVA and Kruskal–Wallis tests were used to determine difference comparisons of three or more groups. The *post hoc* comparisons of ANOVAs, Kruskal–Wallis and log rank test were performed. These results presented in Supplementary Table 10. Chi-square test was used to examine the correlation between m6A modification patterns and clinical features. For each significantly ectopically expressed genes the Kaplan-Meier analysis was performed using the log-rank test. Cox regression analysis of univariate and multivariate variables was used to study the relationship between the prognosis value and different variables. The P values were corrected for multiple comparisons via the Benjamini and Hochberg (BH). Unsupervised subclass mapping method (SubMap) was used to clarify the corresponding relationship of m6A clusters between TCGA-LIHC and ICGC-LIRI cohorts^4^ (Hoshida et al., 2007; Chaisaingmongkol et al., 2017). *P* < 0.05 was considered to have Significant similarity between clusters found by the SubMap method, and this *P* values were corrected by the Bonferroni method. The Tumor Immune Dysfunction and Exclusion (TIDE) were used to calculate TIDE scores and predict the clinical response to immune checkpoint blockade (Şenbabaoǧlu et al., 2016). In all cases, *P* < 0.05 was considered statistically significant.

## Results

### The Landscape of Genetic Variation of m6A Regulators in HCC

**FIGURE 1:**
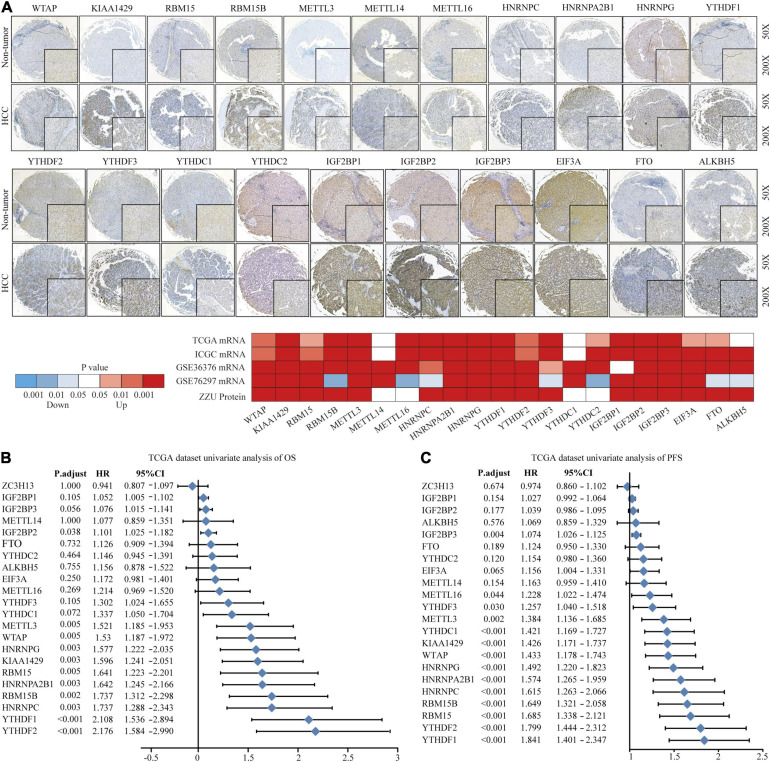
Landscape of genetic variation of m6A regulators in HCC. **(A)** The mRNA and protein expression pattern of m6A regulators in HCC. **(B)** Univariate Cox regression analysis of OS in HCC patients. **(C)** Univariate Cox regression analysis of PFS in HCC patients.

To explore the significant biological function of m6A regulators in hepatocarcinogenesis and tumor progression, we summarized the mRNA and protein expression levels of 22 m6A regulators in HCC and non-tumor tissues based on TCGA, ICGC, GEO, and ZZU TMA cohorts. Both the mRNA and protein expression levels of WTAP, KIAA1429, RBM15, RBM15B, METTL3, HNRNPs, YTHDF1, YTHDF2, YTHDF3, IGF2BPs, and FTO were markedly higher in HCC tissues (Figure 1A). Additionally, to gain insight into the cause of m6A regulator dysregulation, we explored the somatic mutations and copy number variation (CNV) alteration frequency of m6A regulators. Among 364 samples, 35 (9.62%) had m6A regulator mutations, indicating that m6A regulator somatic mutations are infrequent in HCC (Supplementary Figure 2A). The CNV alteration frequency study indicated that CNV alteration was prevalent in m6A regulators. Meanwhile, m6A regulators with amplified CNV (e.g., KIAA1429 and YTHDF1) were markedly upregulated in the HCC tissues (Supplementary Figure 2B). Univariate Cox regression analysis showed that most of the upregulated m6A regulators are potential prognostic risk factors for patients with HCC (Figures 1B,C). Multivariate Cox regression analysis indicated that YTHDF2 was an independent risk factor for OS and progression-free survival (PFS) (Supplementary Figures 2C,D). Correlation analysis indicated that there were higher correlations among m6A regulators (Supplementary Figure 2E and Supplementary Table 5). Overall, the results present large genomic and expression variations of m6A regulators between normal and HCC tissue. Concurrently, the expression of the 22 m6A regulators was closely related, playing a significant role in HCC prognosis.

### Correlation of m6A Clusters With Clinical Features and Prognosis in TCGA Dataset

**FIGURE 2:**
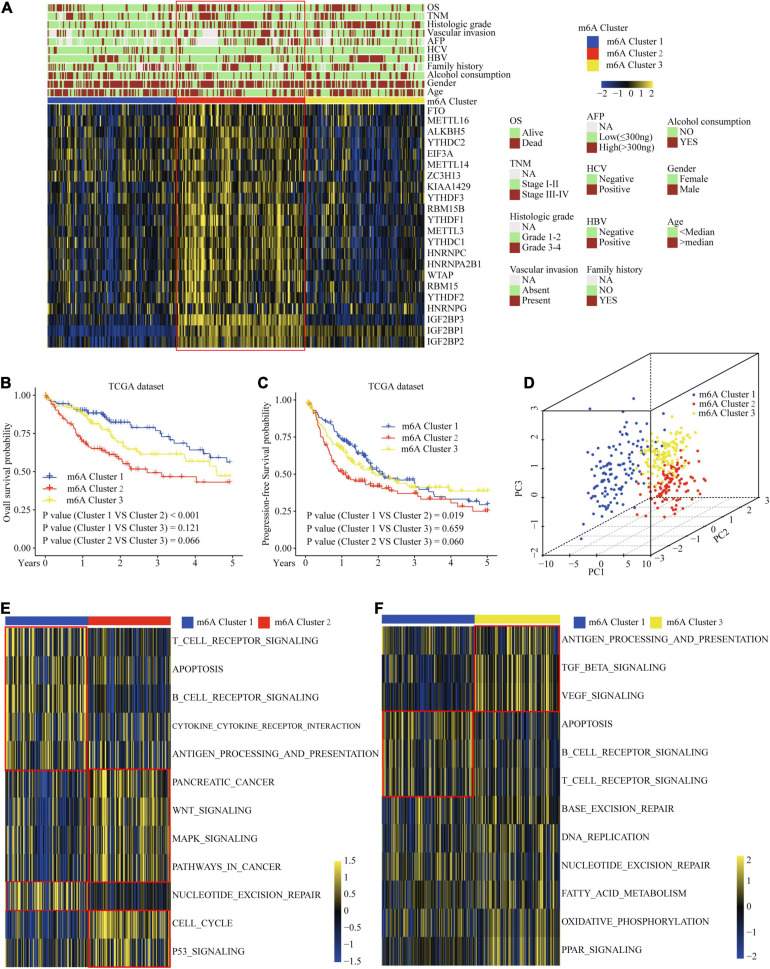
Correlation of the m6A clusters with clinical features, prognosis and biological characteristics in TCGA dataset. **(A)** The relationship between the m6A regulators expression profiles of these three clusters and clinical features of HCC. **(B)** Overall survival analysis for the HCC patients of three clusters in the TCGA dataset. **(C)** Progression-free survival analysis for the HCC patients of three clusters in the TCGA dataset. **(D)** PCA plots of TCGA-LIHC RNA-sequence profiles for three m6A clusters. **(E,F)** GSVA enrichment score showing the activation states of biological pathways in three m6A clusters. Red box indicates the genes expression and clinical features of clusters.

To explore the biological function of different m6A methylation modification patterns in HCC, we performed unsupervised clustering based on the expression of 22 m6A regulators in TCGA-LIHC dataset, and identified three distinct modification clusters. Further analysis of the m6A transcriptional profiles revealed that a significant distinction in three different m6A modification patterns. m6Acluster 1 presented moderate expression in most m6A regulators except for the IGFBPs. m6Acluster 2 was characterized by the increased expression of all m6A regulators. m6Acluster 3 exhibited significant low expression in most m6A regulators except for IGFBP1 and IGFBP2 (Figure 2A). We found that there were significant correlations between clinicopathological features and the m6A clusters. Lack of vascular invasion, low serum alpha-fetoprotein (AFP) level, histologic grade G1/G2, and tumor-node-metastasis (TNM) stage I/II were associated with the C1 or C3 clusters; presence of vascular invasion, advanced TNM stage (III/IV), histologic grade (G3/G4), and high serum AFP level were associated with the C2 cluster (Supplementary Table 6). Prognostic analysis showed the particularly prominent survival advantage in m6Acluster 1, followed by that in m6Acluster 3. m6Acluster 2 had the worst outcome (Figures 2B,C). And the survival advantage of m6Acluster 1 was confirmed in patients with different ages (age ≤ 55 or age > 55) (Supplementary Figure 3). Further, PCA dimension reduction analysis showed that the m6A clusters were segregated into three discrete clusters (Figure 2D). The results suggest that different m6A modifications have significant correlation with HCC clinical characteristics and prognosis.

### Correlation of the m6A Clusters With Tumor Microenvironment (TME) Immune Cell Infiltration Characteristics

**FIGURE 3:**
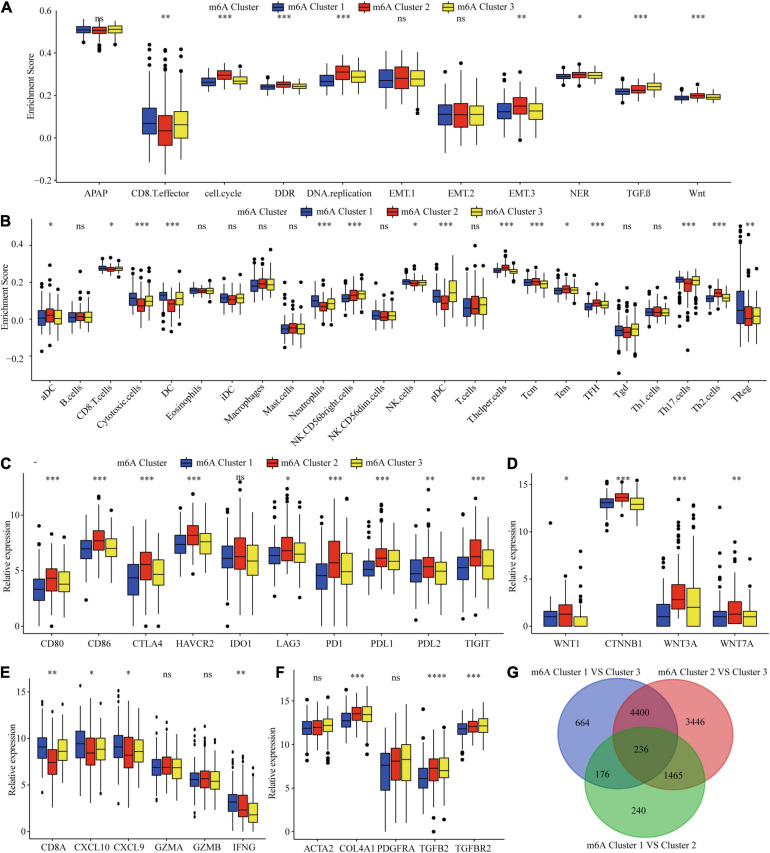
TME immune cells infiltration, biological functions and transcriptome traits in three m6A clusters. **(A)** Difference in biological functions among three m6A clusters in TCGA dataset. **(B)** Difference in the abundance of immune infiltrating cells among three m6A clusters. **(C)** Difference in the immune-checkpoint related genes expression among three m6A clusters. **(D)** Difference in the Wnt pathway related genes expression among three m6A clusters. **(E)** Difference in the immune-activation related genes expression among three m6A clusters. **(F)** Difference in the TGF-β pathway related genes expression among three m6A clusters. **(G)** 236 m6A clusters related genes shown in Venn diagram. ^∗^*P* ≤ 0.05; ^∗∗^*P* ≤ 0.01; ^∗∗∗^*P* ≤ 0.001; ^****^P ≤ 0.0001.

Considering that the classification was based on m6A regulators, we explored whether distinct m6A clusters had different biological behaviors. First, we conducted GSVA analysis. Figures 2E,F and Supplementary Table 7 show that m6Acluster 1 was markedly enriched in cytokine–cytokine receptor interaction, T and B cell receptor signaling pathways, NER, and apoptosis pathways. m6Acluster 2 presented enrichment pathways related to WNT, MAPK, and the cell cycle pathways. m6Acluster 3 was prominently associated with the TGF-β and MAPK signaling pathways. Further immune infiltration and mechanism studies demonstrated that compared with m6Acluster 2, m6Acluster 1 and 3 showed high infiltration of most immune cells, but m6Acluster 3 did not show higher CD8^+^ positive T cell infiltration and significant survival advantage, which may be related to the immunosuppression caused by TGF-β pathway significant enrichment (Figures 3A,B). A surprising finding was that the m6A modification patterns had significantly distinct immune subtypes. m6Acluster 1 was classified as the immune-inflamed phenotype, characterized by adaptive immune cell infiltration. m6Acluster 2 was classified as the immune-desert phenotype, characterized by the inhibition of immunity and WNT pathway significant enrichment. m6Acluster 3 was classified as the immune-excluded phenotype, characterized by innate immune cell infiltration and TGF-β significant enrichment. To investigate the m6A-related immune phenotypes, we extracted pathway- and immune-associated key gene signatures from the published literature. We found that the mRNAs relevant to immune checkpoints and the WNT pathway were significantly upregulated in m6Acluster 2 (Figures 3C,D). The immune activation genes CD8A, CXCL9, and CXCL10 had significant high expression in m6Acluster 1 (Figure 3E), while the TGF-β pathway-related genes exhibited high expression in m6Acluster 3 (Figure 3F). These results demonstrate that there is a close relationship between m6A clusters and TME immune status.

### Correlation of the m6A Clusters With Clinical and TME Cell Infiltration Characteristics in the ICGC Dataset

**FIGURE 4:**
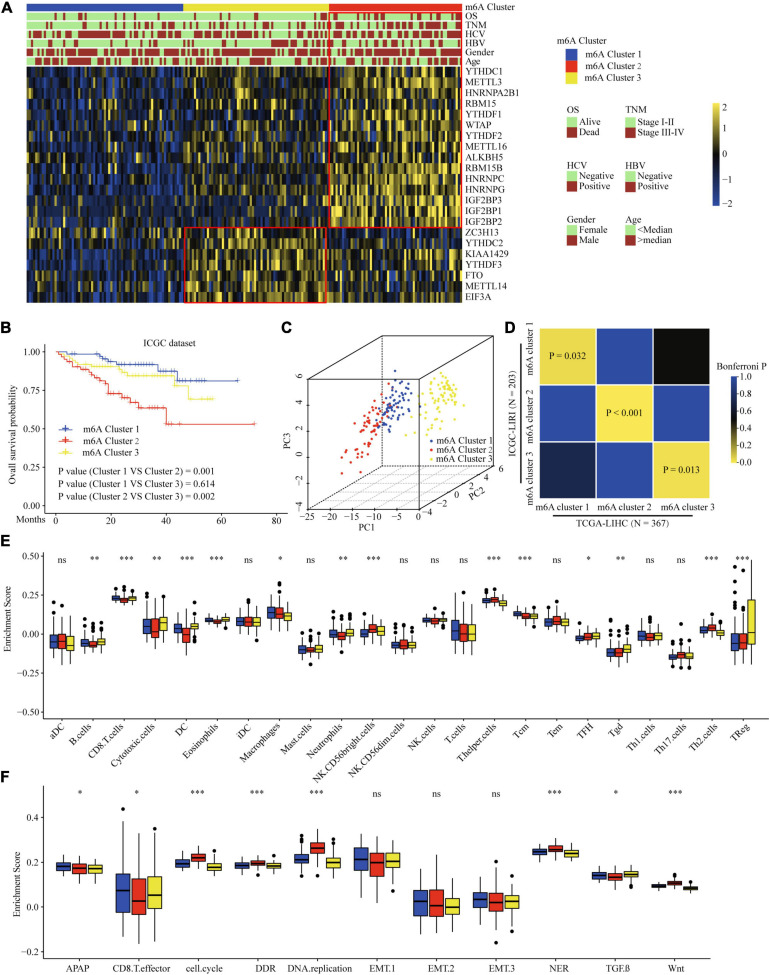
Correlation of the m6A clusters with clinical features, prognosis and biological characteristics in ICGC dataset. **(A)** The relationship between the m6A regulators expression profiles of these three clusters and clinical features of HCC in ICGC dataset. **(B)** Survival analysis for the HCC patients of three clusters in the ICGC dataset. **(C)** PCA plots of ICGC-LIRI-JP RNA-sequence profiles for three m6A clusters. **(D)** Subclass Mapping of TCGA-LIHC and ICGC-LIRI m6A clusters. *P* < 0.05 was considered to have Significant similarity between clusters. **(E)** Difference in the abundance of immune infiltrating cells among three m6A clusters in ICGC dataset. **(F)** Difference in biological functions among three m6A clusters in ICGC dataset. Red box indicates the genes expression and clinical features of clusters. ^∗^*P* ≤ 0.05; ^∗∗^*P* ≤ 0.01; ^∗∗∗^*P* ≤ 0.001.

To validate the correlation of the m6A clusters with the clinical and TME cell infiltration characteristics, we focused on the ICGC cohort for external validation. Similar to TCGA dataset clustering, three fully distinct m6A modification patterns were identified. m6Acluster 1 was characterized by the decreased expression of most of the m6A regulators. m6Acluster 2 showed high expression of YTHDC1, METTL3/16, HNRNPs, RBM15, YTHDF1/2, WTAP, ALKBH5, RBM15B, and IGF2BPs; m6Acluster 3 exhibited significant upregulation of ZC3H13, YTHDC2, YTHDF3, FTO, METTL14, and EIF3A (Figure 4A). Clinical characteristics analysis showed that m6Acluster 2 patients had high serum AFP levels, TNM stage, and were hepatitis B virus (HBV)-positive (Figure 4A and Supplementary Table 8). Prognostic analysis also revealed that m6Acluster 2 was significantly related with poor survival (Figure 4B). PCA dimension reduction analysis visualization of the transcriptomic data of the three m6A clusters showed that they were segregated into three discrete clusters (Figure 4C). To further examine consistency in cluster formation, we used an SubMap method. The SubMap method conducted a pairwise comparison of the molecular features between each of the predetermined m6A clusters of TCGA-LIHC and ICGC-LIRI cohorts. The result showed that the molecular features of m6A clusters between TCGA-LIHC and ICGC-LIRI cohorts are significantly similar (Figure 4D). Further immune infiltration and pathway score analysis indicated that m6Acluster 1 and 3 showed high immune cell infiltration, but that the TGF-β pathway was significantly enriched in m6Acluster 3. m6Acluster 2 presented the lowest level of immune cell infiltration and WNT pathway significant enrichment (Figures 4E,F). The results again confirm the ability of m6A regulators to distinguish different subtypes of HCC.

### Upregulated YTHDF1 Reduced CD3^+^ and CD8^+^ T Cell Infiltration in HCC

**FIGURE 5:**
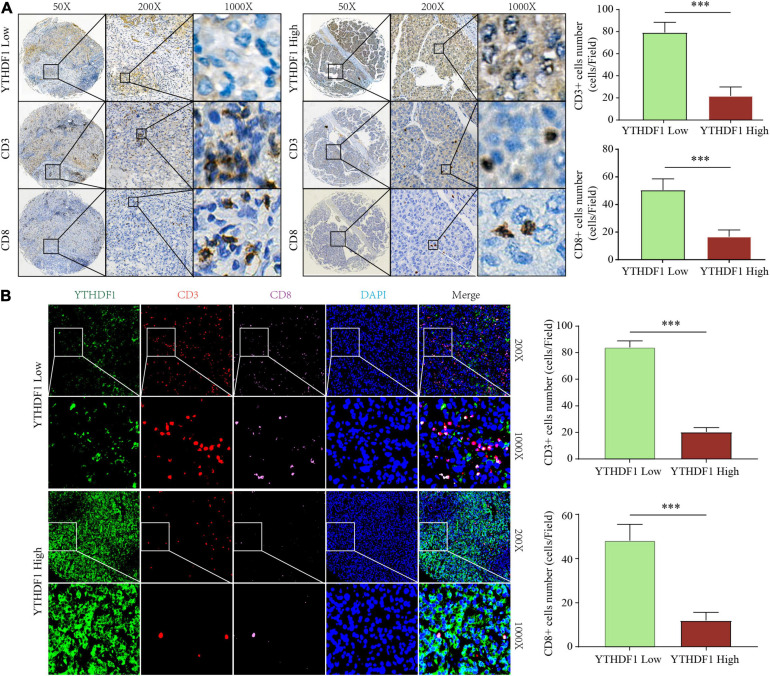
YTHDF1 expression level closely associated with CD3 and CD8 positive T cells infiltration in HCC. **(A)** Immunohistochemistry assays showed that CD3+ and CD8 + T cell density in HCC tissues with high or low YTHDF1 expression. **(B)** immunofluorescent IHC staining of YTHDF1, CD3, and CD8 were performed on TMA-cohort. ^∗∗∗^*P* ≤ 0.001.

The earlier results reveal that different m6A clusters have different immune subtypes. To explore the effect of the expression of the 22 m6A regulators on immune cell infiltration, we first examined the specific correlation between each TME-infiltrating cell type and the 22 regulators using Pearson analyses. We found significantly negative correlations between the level of immune cell infiltration, such as that by B cells, T cells, and CD cells, with the expression of most of the m6A regulators (Supplementary Figure 4A). Subsequently, we found that high YTHDF1 expression was closely related with poor prognosis and infiltration by numerous immune cells (Supplementary Figures 4B–D). Additionally, we explored the effect of YTHDF1 protein level on T cell infiltration. IHC analysis indicated that CD3^+^ and CD8^+^ T cell numbers were significantly decreased in the samples with upregulated YTHDF1 (Figure 5A). To study the essential relationship between TME immune status and YTHDF1 level in patients with HCC, we quantitatively analyzed the CD3^+^ and CD8^+^ T cell counts with immunofluorescence assay. The results demonstrated that YTHDF1 overexpression significantly decreased CD3^+^ and CD8^+^ T cell infiltration (Figure 5B). Based on these findings, it is evident that upregulated YTHDF1 is closely associated with poor prognosis and immune suppression in HCC. Subsequently, pathway enrichment analyses indicated that tumors with low YTHDF1 expression exhibited obvious enhancement in CD8^+^ T effector cells and had inhibited cell cycle, DDR, DNA replication, TGF-β, and WNT pathways (Supplementary Figure 4E).

### m6A Gene Signature Subtypes and m6Ascore Performance Validation

**FIGURE 6:**
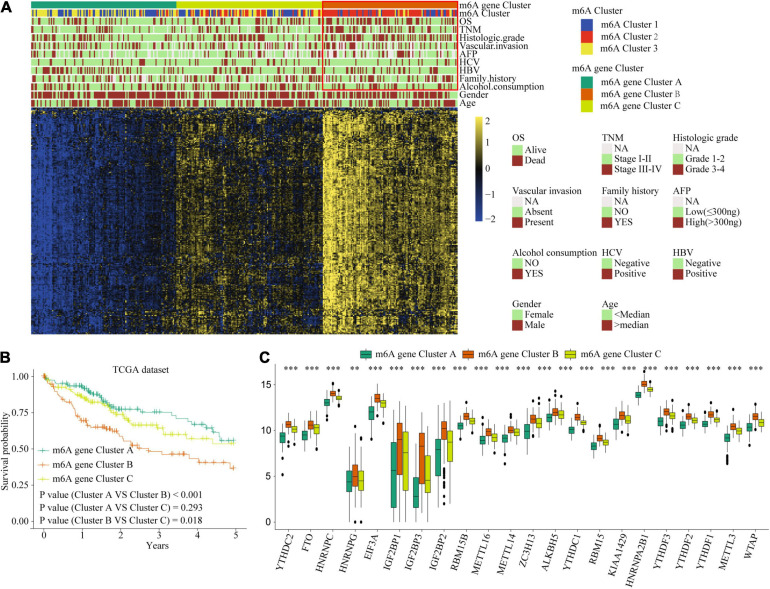
The interrelation of the m6A scores with clinicopathological characteristics and prognostic. **(A)** Unsupervised clustering of 236 m6A related genes in TCGA cohort to classify patients into three m6A gene clusters. **(B)** Survival analysis for the HCC patients of three m6A gene clusters in the TCGA dataset. **(C)** The expression of 22 m6A regulators in three m6A gene clusters. Red box indicates the genes expression and clinical features of clusters. ^∗∗^*P* ≤ 0.01; ^∗∗∗^*P* ≤ 0.001.

Considering the variation and biological function of m6A modification in HCC, we explored the potential biological function of each m6A modification pattern. Differential analysis and a Venn diagram showed that there were 236 common differential genes among the three m6A clusters (Figure 3G). Unsupervised clustering analyses based on the 236 genes confirmed that there were three distinct m6A modification genomic phenotypes; we termed these three clusters m6A gene cluster A–C (Figure 6A). Clinical features analysis indicated that m6A gene cluster B exhibited more vascular invasion, AFP elevation, high histologic grade, and TNM stage (Supplementary Table 9). Prognostic analysis demonstrated a particularly prominent survival advantage in the m6A gene cluster A modification pattern, followed by that of m6A gene cluster C. m6A gene cluster B had the worst outcome (Figures 6B,C). The results again show that m6A methylation patterns are tightly associated with HCC development and progression.

**FIGURE 7:**
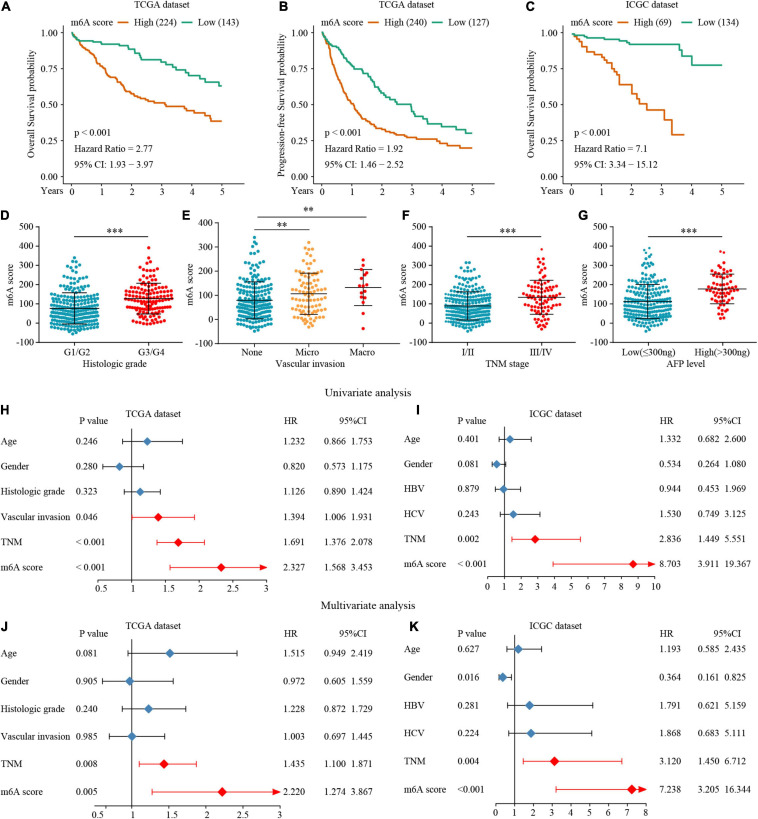
The interrelation of the m6A scores with clinicopathological characteristics and prognosis. **(A–C)** Survival analysis for the HCC patients of m6A scores in the TCGA and ICGC dataset. **(D–G)** The relationship between the m6A scores and clinical characters. **(H–K)** The Univariate and multivariate Cox regression analyses of m6A scores in TCGA and ICGC datasets. ^∗∗^*P* ≤ 0.01; ^∗∗∗^*P* ≤ 0.001.

To accurately evaluate the m6A methylation modification of individual patients with HCC, we selected 182 differential genes with prognostic utility to construct the patients’ individual m6Ascores (Supplementary Table 11). To obtain the clinical and prognostic value for the patients with HCC, the best cut-off value was calculated with the survminer package, and the patients were divided into low or high m6Ascore groups. A high m6Ascore indicated worse prognosis (Figures 7A,B). Meanwhile, validation in an external ICGC database confirmed the prognostic value of the m6Ascore (Figure 7C). Thereafter, we quantitatively analyzed the m6Ascore in HCC to investigate the association between the m6Ascore and each clinicopathological characteristic. Figures 7D–G shows that the m6Ascores were significantly different in these groups, with TCGA dataset compartmentalized by histologic grade, vascular invasion, TNM stage, and AFP level. Univariate and multivariate Cox regression analyses were performed with TCGA and ICGC datasets. The m6Ascore was an independent prognostic factor for HCC outcome (Figures 7H–K).

**FIGURE 8:**
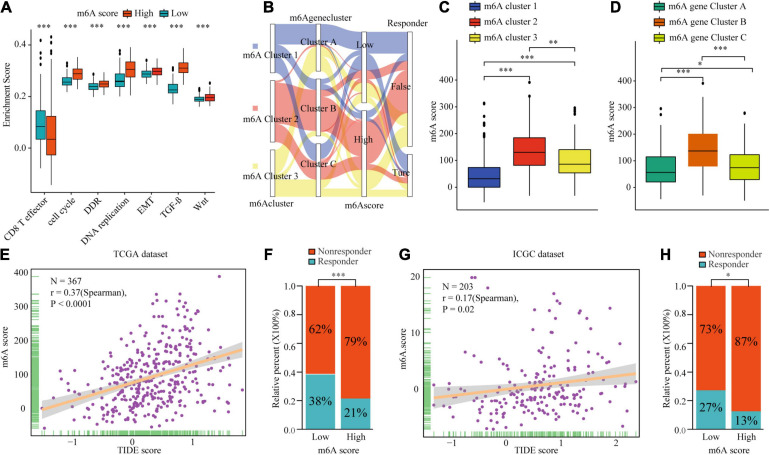
The biological mechanism and immunotherapy value of m6Ascore. **(A)** Difference of biological functions between m6A score high and low. **(B)** Alluvial diagram showing the changes of m6A clusters, m6A gene clusters, m6A scores, and respond to immunotherapy. **(C)** Differences in m6Ascore among three m6A clusters in TCGA cohort. **(D)** Differences in m6Ascore among three m6A gene clusters in TCGA cohort. **(E–H)** Correlation analysis of m6A scores and TIDE scores and the proportion of patients with response to immunotherapy in low or high m6Ascore groups in TCGA dataset **(E,F)** and ICGC dataset **(G,H)**. ^∗^*P* ≤ 0.05; ^∗∗^*P* ≤ 0.01; ^∗∗∗^*P* ≤ 0.001.

To explore the potential biological mechanism of the m6Ascore, we tested the correlation between it and the known pathway signatures. The results indicated that a low m6Ascore could be significantly associated with CD8^+^ T cell effector, whereas a high m6Ascore could be linked to significant enrichment of the immunosuppression and malignant progression pathways (Figure 8A). Furthermore, we explored the relationship among m6A modification, m6Ascore, and HCC immunotherapy. Differential analysis found that m6Acluster 1 and m6A gene cluster B had the highest m6Ascores, while m6Acluster 1 and m6A gene cluster B had the lowest m6Ascores (Figures 8B–D). Then, we used the TIDE algorithm to predict the likelihood of response to immunotherapy based on TCGA and ICGC datasets. A previous study had demonstrated that a higher TIDE score indicated worse immunotherapy response. Correlation analysis showed a significantly positive correlation between the m6Ascore and the TIDE score (Figures 8E,G). Meanwhile, we were very delighted to see that patients with low m6Ascores had more promising to response to immunotherapy (Figures 8F,H). Overall, our study indicates that the m6Ascore might be a potential biomarker for evaluating the immunotherapy effect and prognosis in HCC.

## Discussion

Hepatocellular carcinoma is one of the most frequently diagnosed malignancies worldwide, with poor prognosis (Dominissini et al., 2012; Meyer et al., 2012). Hence, there is an urgent need to identify powerful diagnostic and novel therapeutic strategies to improve HCC diagnosis and treatment. Numerous studies have demonstrated that harnessing the immune system against cancer has become an effective therapy option (Makarova-Rusher et al., 2015; Topalian et al., 2020). Recent clinical studies have verified that the PD-1 inhibitor nivolumab has raised hope for the successful treatment of advanced HCC (Yau et al., 2020; Kim et al., 2021). However, a small proportion of patients with HCC can benefit from immune checkpoint inhibitor therapy. Therefore, identifying novel biomarkers would allow better patient selection for individual immune and targeted therapy.

Previously studies have demonstrated that m6A modification plays a critical role in HCC progression and the shaping of TME, e.g., YTHDF1 promotes tumor progression and was closely associated with poor prognosis (Wang T. et al., 2020). Meanwhile, the study of Han et al. (2019) revealed that inhibition of YTHDF1 strengthened the ability of tumor APAP in DCs, which in turn enhanced tumor infiltrating CD8 + T cell antitumor response. YTHDF2 regulates mRNA degradation by recognizing mRNA m6A sites, and facilitates the proliferation of HCC cells (Yang et al., 2017; Zhang C. et al., 2020). In parallel, it was found that YTHDF2 suppress inflammation and angiogenesis in the tumor cell hypoxia environment (Hou et al., 2019). METTL3 enhances HCC cell growth ability (Liu et al., 2020; Yang et al., 2021). METTL14 suppresses the metastatic potential of HCC by modulating m6A-dependent tumor-suppressor primary miRNA processing (Ma et al., 2017; Shi et al., 2020). Wang et al. (2019) reported that upregulated METTL3 promoted DC activation and maturation. METTL3 downregulation inhibited T cell activation and aggregation though downregulation of co-stimulatory molecules CD80 and CD40 (Liu Y. et al., 2019). However, the specific depletion of METTL3 or METTL14 improved the therapeutic efficacy of anti-PDL1 blockade (Wang L. et al., 2020). As most studies focused on single m6A regulators or analyzed public datasets only, a comprehensive and systematic study of the biological function of m6A regulator-associated modification patterns in HCC is necessary.

In the present study, we explored the m6A regulators of mRNA and protein levels based on TCGA and TMA cohorts. The survival analysis clarified the m6A-related regulator effects on the prognoses of the patients with HCC. Furthermore, three distinct m6A clusters were identified based on 22 m6A regulators. The three clusters had significantly distinct prognosis value, clinical features, immune cell infiltration, and pathway signatures. m6Acluster 1 was characterized by the significant enrichment of adaptive immunity pathways, corresponding to the immune-inflamed phenotype, m6Acluster 2 was characterized by the suppression of immunity and WNT pathway activation, corresponding to the immune-desert phenotype, and m6Acluster 3 was classified as the immune-excluded phenotype, characterized by innate immune cell infiltration and TGF-β significant enrichment. The immune-excluded and immune-desert phenotypes could be considered cold tumors. It has been indicated that the activation of WNT–β-catenin signaling mediates T cell exclusion in HCC. Further, the TGF-β pathway suppresses the effect of CD8^+^ T cells by regulating regulatory T cells (Tregs). Mechanistically, previous study showed that m6A modification directly or indirectly involved in the regulation of cancer-related pathways such as proliferation, apoptosis, invasion and metastasis, and metabolic reprogramming (Li et al., 2021). Some investigators have found that YTHDF1 regulated the translation of FZD7 which is a key Wnt receptor by an m6A-dependent manner (Pi et al., 2021). The m6A modification of CTNNB1 promotes the expression of β-catenin and activates the Wnt pathway (Liu L. et al., 2019). Additionally, the upregulated of TCF1 regulated by IGF2BP2-mediated m6A modification activates the Wnt pathway and the expression of the downstream effector molecules (Wang K. et al., 2020). The m6A modification of the 5′-UTR and coding sequence (CDS) regions of TGF-β promotes the degradation of mRNA encoding TGF-β and thereby inhibits the TGF-β signaling pathway (Li et al., 2020a). METTL3 contributes to TGF-β induced epithelial-mesenchymal transition through the regulation of JUNB in lung cancer (Wanna-Udom et al., 2020). The immune-inflamed phenotype, known as hot tumor, demonstrates a large amount of immune cell infiltration in the TME. Consistent results were confirmed in both TCGA and ICGC datasets. The consistency of immune cell infiltration characteristics and pathway signatures confirmed the reliability of our immunophenotype classification for the different m6A clusters.

Next, we identified 236 differential genes in three distinct m6A clusters. These differential genes were considered m6A cluster-related genes. Similar to the m6A clusters, three m6A modification genomic phenotypes were identified based on the m6A cluster-related genes. Clinical features and prognosis analyses indicated that the m6A methylation pattern is tightly associated with HCC development and progression. Considering the high degree of m6A modification heterogeneity, 182 differential genes with prognostic utility were selected to construct the m6Ascores of individual patients. Patients with high m6Ascores demonstrated worse prognosis and clinical features. Meanwhile, high m6Ascores indicated significant enrichment of the cell proliferation, WNT, and TGF-β pathways, and the inhibition of CD8^+^ T effector cells. The m6A subtype characterized by the immune-excluded phenotype exhibited a higher m6Ascore, while the pattern characterized by the immune-inflamed phenotype showed a lower m6Ascore. Additionally, TIDE analysis showed that the m6Ascore had a predictive advantage in immunotherapy for HCC. Generally, the m6A scores were closely associated with immune cell infiltration and could be used as prognostic markers for HCC. To date, there are some studies have analyzed the relationship among m6A modification patterns, m6A scores, tumor progression, and immune cell infiltration in many solid malignancies. Consistently, Zhang B. et al. (2020) and Chong et al. (2021) identified three different m6A subtypes according to the expression of m6A regulators in colon and gastric cancer. After comprehensively evaluated the association among immune cell infiltration, prognosis, and pathway scores, three m6A patterns to different immune phenotypes (immune-inflamed, immune-excluded, and immune-desert) were constructed. Then m6A score calculated based on the m6A modification, were closely associated with tumor progression, prognosis, immune infiltration subtypes and immunotherapy response in colon cancer and gastric cancer.

Our data also reveal that YTHDF1 plays an important role in the development and immune response of HCC. We found significantly negative correlations between the level of immune cell infiltration such as that by B cells, T cells, and CD cells with the expression of most of the m6A regulators. Subsequently, we focused on YTHDF1. Han et al. (2019) demonstrated that *Ythdf1*-deficient mice exhibit an elevated antigen-specific CD8^+^ T cell anti-tumor response because suppressing YTHDF1 in the DCs enhanced the cross-presentation of tumor antigen and the cross-priming of CD8^+^ T cells *in vivo*. However, the immunomodulatory function of YTHDF1 dysregulation in HCC cells is unclear. In the present study, IHC and immunofluorescence demonstrated that YTHDF1 overexpression significantly decreased CD3^+^ and CD8^+^ T cell infiltration in HCC. Meanwhile, patients with high YTHDF1 expression exhibited obvious TGF-β and WNT pathway enhancement. These results indicate that YTHDF1 might induce immunosuppression by activating the TGF-β and WNT pathways. Our findings provide novel ideas for promoting personalized cancer immunotherapy and potential therapeutic targets for HCC.

## Conclusion

We show that m6A modification patterns play a crucial role in the tumor immune microenvironment and prognosis of HCC. Upregulated YTHDF1 mediates m6A modification, playing a critical role in suppressing anti-tumor immune responses.

## Data Availability Statement

The datasets presented in this study can be found in online repositories. The names of the repository/repositories and accession number(s) can be found in the article/Supplementary Material.

## Ethics Statement

This study was approved by the Institutional Review Board of the First Affiliated Hospital of Zhengzhou University. Written informed consent for participation was not required for this study in accordance with the national legislation and the institutional requirements.

## Author Contributions

JL, YZ, and WW performed all the experimental work. LL, XL, MH, and GZ completed the data collection. GC, KG, and XC participated in data analysis. RS and LL conceived and participated in the design of the study. JL and RS wrote the manuscript. All authors read and approved the final manuscript.

## Conflict of Interest

The authors declare that the research was conducted in the absence of any commercial or financial relationships that could be construed as a potential conflict of interest.

## Footnotes

1 https://tcga-data.nci.nih.gov/tcga/

2 https://icgc.org/

3 http://www.ncbi.nlm.nih.gov/geo/

4 http://genepattern.broadinstitute.org/
