# Supplementary material for: m6A Regulator-Associated Modification Patterns and Immune Infiltration of the Tumor Microenvironment in Hepatocarcinoma
Source: Front Cell Dev Biol. 2021 Jul 2;9:687756. doi: 10.3389/fcell.2021.687756 (PMC8283020; doi:10.3389/fcell.2021.687756)

**Figure S1: Overview of this study design.**

**
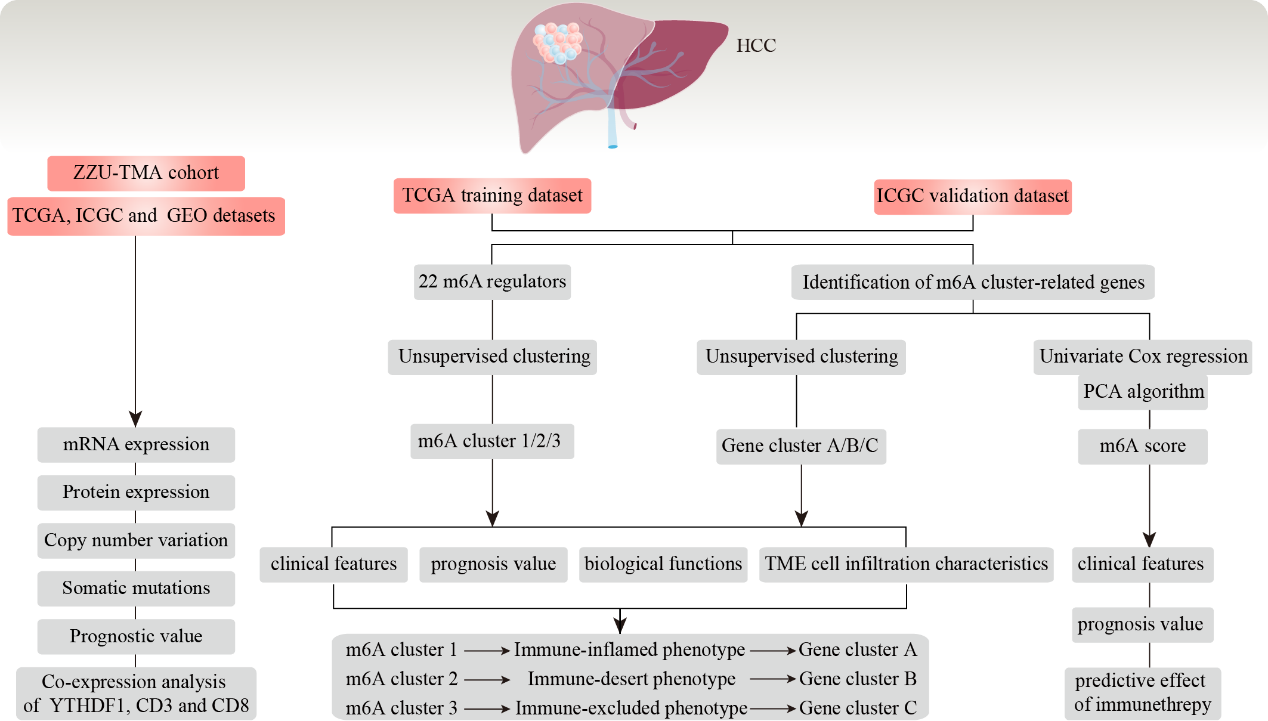
**

**Figure S2: The mutation, CNV variation frequency, prognosis value and correlation of 22 m6A regulators in TCGA-LIHC cohort. (A)** The mutation frequency of 22 m6A regulators in TCGA-LIHC cohort. **(B)** The CNV variation frequency of 22 m6A regulators in TCGA-LIHC cohort. **(C)** Multivariate Cox regression analysis of overall survival in HCC patients. **(D)** Multivariate Cox regression analysis of Progression-free survival in HCC patients. **(E)** Pearson correlation analysis of the 22 m6A regulators.


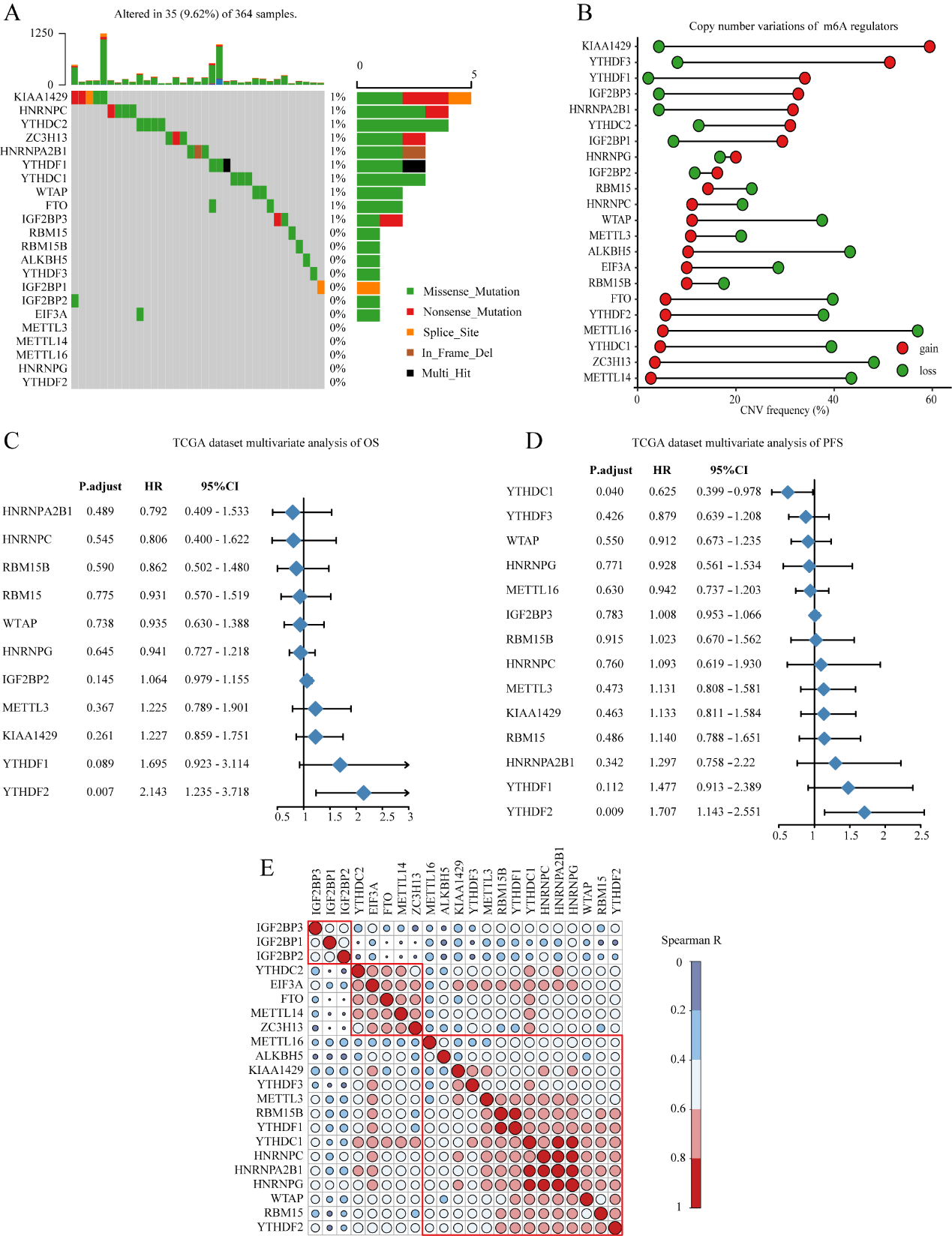


**Figure S3: Kaplan-Meier overall survival curves of m6A clusters for patients in the TCGA dataset stratified by age. (A-B)** Kaplan-Meier OS and PFS survival curves for patients (age ≤ 55 years) in the TCGA dataset. **(C-D)** Kaplan-Meier OS and PFS survival curves for patients (age > 55 years) in the TCGA dataset.

**
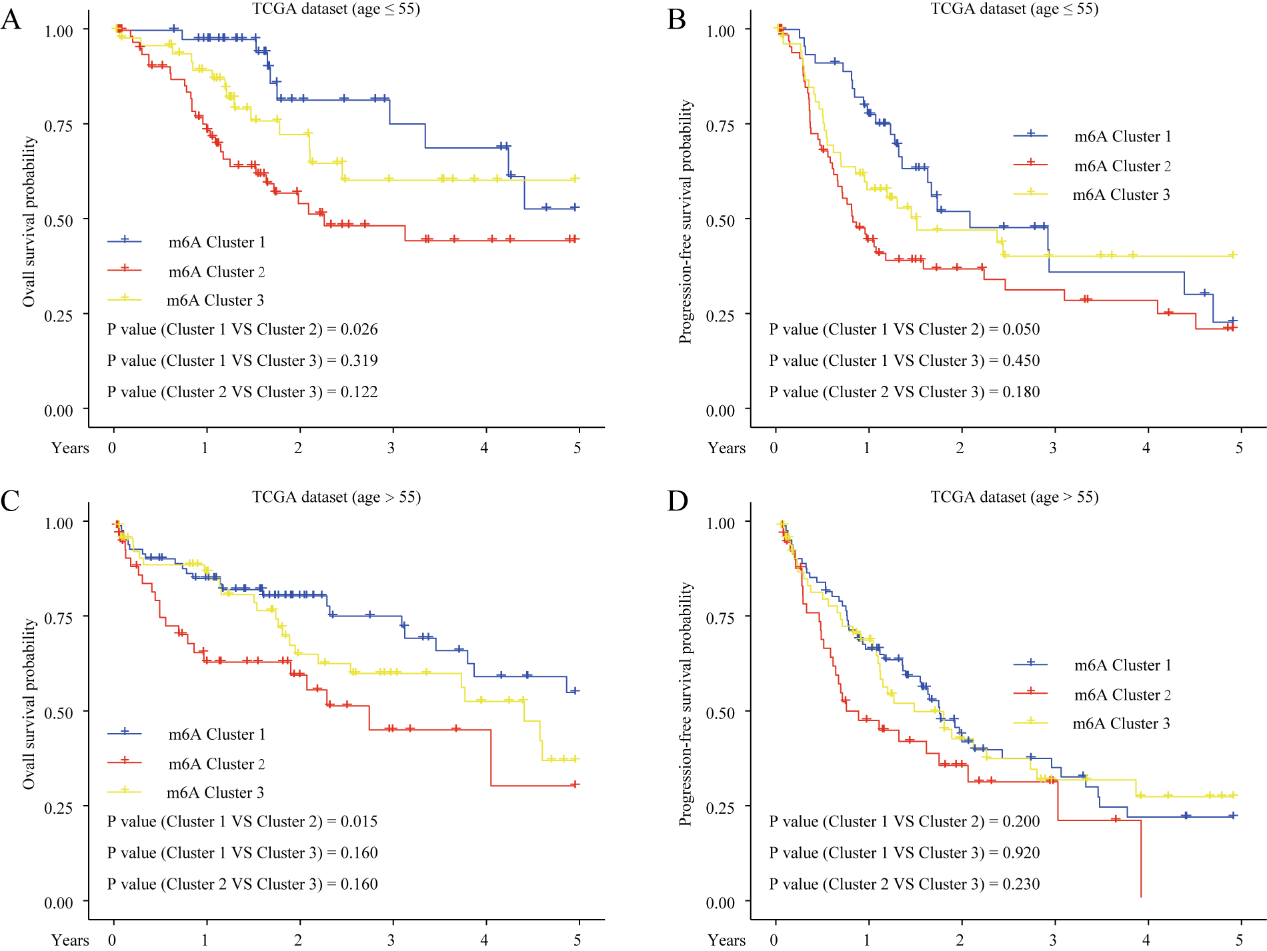
**

**Figure S4: Correlation between m6A regulators expression and TME immune cells infiltration and the roles of YTHDF1 in TME. (A)** The correlation between each TME infiltration cell type and each m6A regulator using spearman analyses. **(B)** Difference in TME infiltration cell between YTHDF1 high expression and low expression. **(C-D)** Survival analyses for patients with low or high YTHDF1 expression in TCGA cohort. **(E)** Differences in key pathways between YTHDF1 high expression and low expression groups.


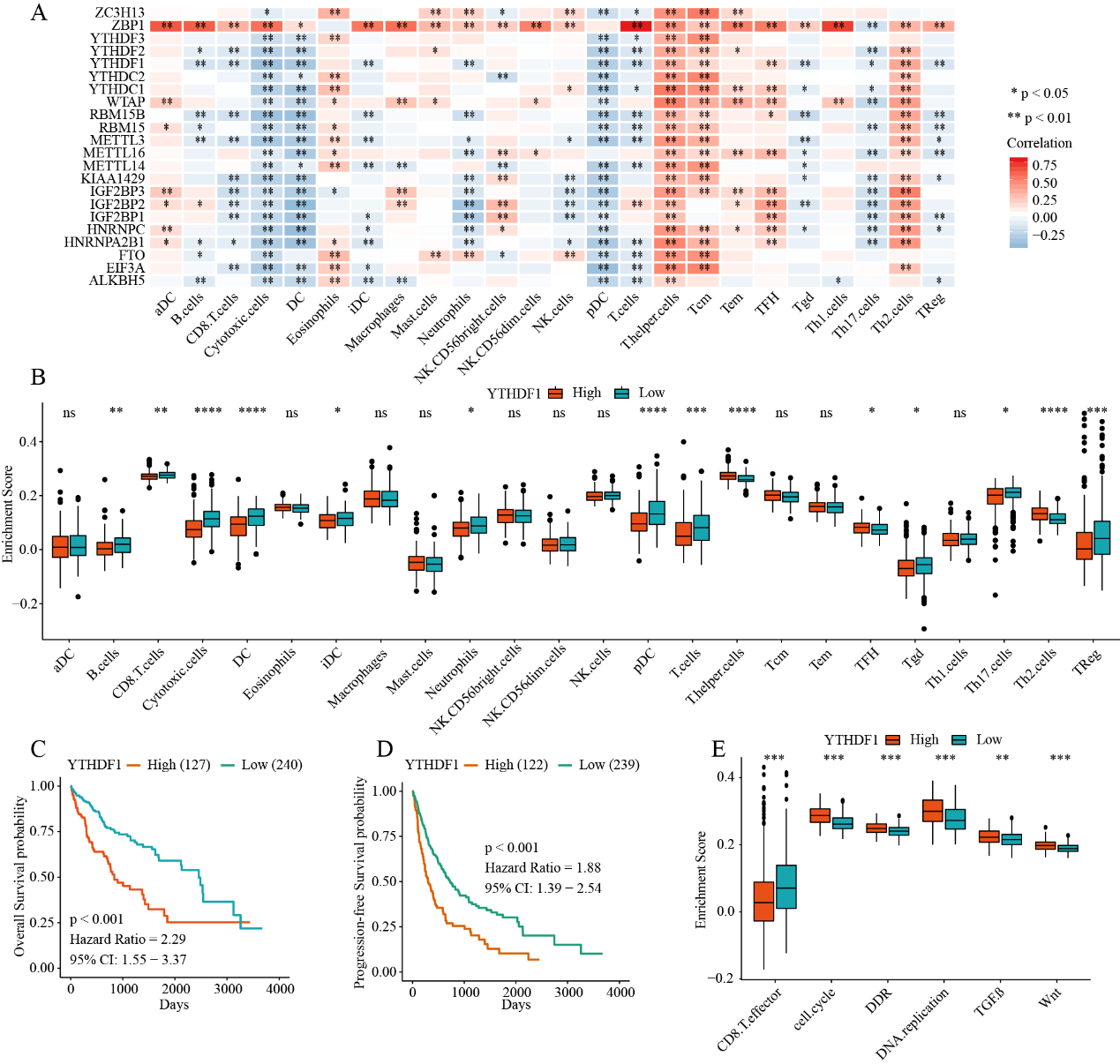

Supplement: Supplementary file 1 [file Table_1.docx]
